# Supplementary material for: Chromothripsis during telomere crisis is independent of NHEJ, and consistent with a replicative origin
Source: Genome Res. 2019 May;29(5):737–49. doi: 10.1101/gr.240705.118 (PMC6499312; doi:10.1101/gr.240705.118)
Supplement: Supplemental Material [file supp_gr.240705.118_Supplemental_file_1.zip › contigs/annotated_contigs/DB50/contig.2.DB50_length_663_mean_cov_10.2911010558.docx]

**DB50_length_663_mean_cov_10.2911010558**

```
AATAATATACTCTCTTCAAAAATGTACTTAAGATGTATACATAAAGTAAATGGATGTCAAAGATATACATTGCAAGCACTTATCCAAAA
 >chr5:27272167-27272500 - E=1e-188
GAACCTTGAGTAGTTATATTAACACAGTGTATACTGACAGCAAGTAATATTGCCAGAAAAAAAATCACTAAATTACATTATGATGAAGG

GGTCAATTCTCCATGAGAACACAGAATCCTAAATATGTATACATCCACCAAAAGAGTGTCAAAATGCTTGAAGCAAAAATTGATAGAAC

TGAAAGGACAAACTATAAAATTAGTAGAATATAGAAAACCTGAATAACGCTATAAAACACC|TTGTT|TGAGACATTACTAGAAGTGGA
                                                              >chr5:27270882-27271217 -
ATTTCTATGTCAAATATTTATTAGGTTAAACACTTTAACATATGTGCCAAATTGCCCCCCTCAAAAAAAGTACCATTATATCTTTTTCT
 E=8e-190
AAAAATATATGAATGTCTATATCCCAGATTCTAGTCAACCTGCAAATTATCAGTTTGAAATATAATGTTAGGGAAGGTCTCATGGGCAT

GTCAGAGAGGTCAGCGTTGTACAGAAACTCACAGGTTTGATCTTACTTGTGCCACTATACTATAGTAATTCTTAAAAGAGAGAAAGAGC

TTGAACATCAGGTAAGTAATGTACTGATTTTGAACCATTATA
```
